# Lifetime Smoking and Asthma: A Mendelian Randomization Study

**DOI:** 10.3389/fgene.2020.00769

**Published:** 2020-08-04

**Authors:** Ming Shen, Xin Liu, Guoqi Li, Zhun Li, Hongyu Zhou

**Affiliations:** Respiratory Hospital of Angang General Hospital, Anshan, China

**Keywords:** asthma, allergic diseases, genome-wide association study, Mendelian randomization, inverse-variance weighted meta-analysis

## Abstract

Evidence from clinical and epidemiological studies indicates that asthma is associated with allergic diseases including hay fever, allergic rhinitis, and eczema. Genetic analysis demonstrated that asthma had a positive genetic correlation with allergic diseases. A Mendelian randomization (MR) analysis using the rs16969968 single-nucleotide variant as the instrumental variable indicated that smoking was associated with increased risk of asthma. However, in a different MR analysis, smoking was significantly associated with reduced hay fever and reduced allergic sensitization risk. These findings revealed inconsistencies in the association of smoking with asthma and allergic diseases. Hence, we conducted an updated MR analysis to investigate the causal association between lifetime smoking and asthma risk by using 124 genetic variants as the instrumental variables. No significant pleiotropy was detected using the MR–Egger intercept test. We found that increased lifetime smoking was significantly associated with decreased asthma risk by using the inverse variance weighted (IVW) method (OR = 0.97, 95% CI 0.956–0.986, and *P* = 1.77E-04), the weighted median regression method (OR = 0.976, 95% CI 0.96–0.994, and *P* = 8.00E-03), and the MR–Egger method (OR = 0.919, 95% CI 0.847–0.998, and *P* = 4.5E-02). Importantly, MR pleiotropy residual sum and outlier (MR-PRESSO) MR analysis also indicated a significant association between increased lifetime smoking and decreased asthma risk with OR = 0.971, 95% CI 0.956–0.986, and *P* = 2.69E-04. After the outlier was removed, MR-PRESSO outlier test further supported the significant association with OR = 0.971, 95% CI 0.959–0.984, *P* = 1.57E-05.

## Introduction

Evidence from clinical and epidemiological studies indicates that asthma is associated with allergic diseases including hay fever, allergic rhinitis, and eczema (Zhu et al., 2018). In 2018, Zhu et al. (2018) used the asthma and allergic disease genome-wide association study (GWAS) datasets including 33,593 cases and 76,768 controls of European ancestry from the UK Biobank and conducted a genetic correlation analysis using the linkage disequilibrium score regression (LDSC) method. They found that asthma had a positive genetic correlation with allergic diseases, which indicated shared genetic etiology (Zhu et al., 2018). In 2017, Ferreira et al. conducted a GWAS analysis of 360,838 samples with kinds of allergic disease phenotypes (Ferreira et al., 2017). They demonstrated that asthma, hay fever, and eczema share many common genetic variants (Ferreira et al., 2017). Importantly, these risk variants could regulate and dysregulate the expression of immune-related genes (Ferreira et al., 2017).

Many observational studies have been conducted to evaluate the association between smoking and asthma risk, but these studies have produced inconsistent findings for this association (Skaaby et al., 2017). Mendelian randomization (MR) analysis methods have been used widely to infer causal associations (Liu et al., 2018; Luo et al., 2019; Zhao and Schooling, 2019; Zhao et al., 2019; Zhuang et al., 2019a, b). Skaaby et al. (2017) used the rs16969968 and rs1051730 single-nucleotide variants, which were in linkage disequilibrium with each other, to genotype 231,020 individuals by observational and MR analyses. The observational analysis revealed similar asthma risk in current smokers and never smokers, and the MR analysis showed that current smokers had higher risk of asthma than never smokers (Skaaby et al., 2017).

Other MR studies using rs16969968 as an instrumental variable for smoking heaviness have reported its association with other human habits, such as coffee consumption (Bjorngaard et al., 2017) and alcohol use (Taylor et al., 2018), and factors such as depression and anxiety (Taylor et al., 2014), and blood pressure and resting heart rate (Linneberg et al., 2015). MR studies that use a single genetic variant as the instrumental variable have some limitations (Rosa et al., 2019). Therefore, an MR analysis using multiple significant and independent genetic variants as potential instrumental variables may increase the statistical power and precision, as was found in some recent studies (Relton and Davey Smith, 2015; Davies et al., 2018; Rosa et al., 2019).

Wootton et al. (2019) performed a GWAS of lifetime smoking behavior (including smoking duration, heaviness, and cessation) by analyzing a total of 462,690 samples from the UK Biobank. They identified 124 genetic variants associated with lifetime smoking that met a genome-wide level of significance of *P* < 5 × 10^–8^ (Wootton et al., 2019). Zhu et al. (2018) conducted a GWAS of asthma with 14,085 asthma subjects and 76,768 controls of European ancestry. We used these lifetime smoking and asthma GWAS datasets to perform an updated MR analysis to evaluate the causal association between lifetime smoking and asthma risk.

## Materials and Methods

### Lifetime Smoking Genome-Wide Association Study Dataset

The lifetime smoking GWAS dataset from the UK Biobank included 462,690 samples from individuals of European ancestry (Wootton et al., 2019). The genetic variant genotype dataset met the genotype quality control criteria (Wootton et al., 2019). The 462,690 samples were from 138,807 smokers and 323,883 controls. Among the 138,807 samples from smokers, 8% were from current smokers and 22% were from former smokers (Wootton et al., 2019). A lifetime smoking measure was used to evaluate smoking initiation, smoking heaviness, and smoking duration (Wootton et al., 2019). We selected the 124 genetic variants associated with lifetime smoking reported by Wootton et al. that met the genome-wide level of significance as the potential instrumental variables (Table 1; Wootton et al., 2019).

**TABLE 1:** 117 genetic variants associated with lifetime smoking and asthma.

SNP	CHR	BP	EA	NEA	EAF	Lifetime smoking GWAS			Asthma GWAS		
							
						Beta	SE	*P* value	Beta	SE	*P* value
rs10282292	7	111092478	C	T	0.362	0.013	0.002	7.10E-10	–0.0004	0.0002	9.73E-01
rs1050847	16	87443734	C	T	0.426	0.011	0.002	1.80E-08	–0.0002	0.0001	9.84E-01
rs10516044	5	167604236	G	C	0.884	–0.02	0.003	2.00E-10	0.0090	0.0452	5.79E-01
rs10879871	12	75380511	T	G	0.343	–0.014	0.002	5.60E-11	0.0050	0.0120	6.61E-01
rs10918701	1	162090536	G	A	0.372	0.012	0.002	2.00E-08	0.0050	0.0149	6.31E-01
rs10922907	1	91193049	A	T	0.451	0.015	0.002	4.40E-13	–0.0045	0.0107	6.64E-01
rs111243290	9	108975927	T	C	0.963	–0.029	0.005	4.30E-08	–0.0119	0.0260	6.77E-01
rs1118814	1	99516611	G	A	0.324	0.012	0.002	1.40E-08	–0.0270	0.0122	1.38E-02
rs11210229	1	73860028	A	G	0.384	0.017	0.002	2.80E-16	0.0080	0.1226	4.74E-01
rs112282219	11	46632809	G	A	0.959	–0.033	0.005	3.70E-11	0.0398	0.0395	1.57E-01
rs11255908	10	8802912	T	G	0.743	–0.015	0.002	1.90E-10	–0.0178	0.0152	1.21E-01
rs113382419	9	136463019	C	A	0.889	–0.041	0.003	9.70E-38	–0.0050	0.0068	7.70E-01
rs1149331	1	7528669	G	A	0.477	–0.011	0.002	3.20E-08	0.0090	0.0299	3.82E-01
rs11783093	8	27425349	C	T	0.839	0.023	0.003	1.00E-16	–0.0010	0.0006	9.65E-01
rs11861214	16	746611	G	T	0.784	0.013	0.002	2.60E-08	0.0175	0.0177	1.62E-01
rs11948770	5	13246336	T	C	0.768	–0.015	0.002	4.00E-10	0.0011	0.0008	9.28E-01
rs1221148	9	122046875	C	G	0.587	0.013	0.002	4.60E-11	–0.0129	0.0163	2.14E-01
rs12244388	10	104640052	G	A	0.661	–0.019	0.002	9.60E-20	0.0178	0.0140	1.02E-01
rs1246265	9	86761745	T	C	0.305	–0.013	0.002	5.30E-09	0.0040	0.0061	7.44E-01
rs12481282	20	44761377	G	C	0.722	–0.013	0.002	7.80E-09	0.0200	0.0142	7.98E-02
rs12623702	2	202885506	A	G	0.613	–0.014	0.002	5.70E-12	0.0130	0.0171	2.24E-01
rs12708665	16	24728227	A	G	0.285	–0.013	0.002	3.40E-09	0.0315	0.0126	6.10E-03
rs12831617	12	84758368	C	T	0.764	–0.013	0.002	2.40E-08	–0.0080	0.0887	5.36E-01
rs12967855	18	35138245	A	G	0.331	0.012	0.002	4.00E-08	–0.0143	0.0168	1.98E-01
rs13009008	2	174043233	A	G	0.328	0.012	0.002	6.40E-09	–0.0106	0.0247	3.35E-01
rs13296519	9	128471924	G	T	0.606	–0.014	0.002	1.40E-11	0.0090	0.0325	3.90E-01
rs136233	22	31212410	A	G	0.809	–0.014	0.003	2.70E-08	0.0107	0.0494	4.15E-01
rs147412694	21	40702786	G	A	0.85	–0.017	0.003	2.30E-09	–0.0227	0.0202	1.31E-01
rs17309874	11	27667236	G	A	0.74	–0.016	0.002	5.60E-13	0.0053	0.0140	6.48E-01
rs17553262	10	92912773	A	C	0.885	–0.018	0.003	6.90E-09	–0.0109	0.3009	4.86E-01
rs17576594	4	147952241	G	A	0.724	0.016	0.002	1.90E-12	0.0065	0.0355	5.73E-01
rs1827535	11	16380409	T	G	0.686	–0.012	0.002	1.20E-08	0.0109	0.0244	3.28E-01
rs1922018	7	3560401	C	T	0.364	0.014	0.002	2.50E-12	–0.0013	0.0010	9.04E-01
rs1931263	1	96175101	G	T	0.51	–0.011	0.002	4.00E-08	0.0087	0.0327	3.95E-01
rs1933270	1	49977965	T	G	0.364	0.013	0.002	1.70E-10	–0.0086	0.0417	4.18E-01
rs202645	22	41798520	A	G	0.203	–0.015	0.002	5.00E-09	0.0564	0.0131	8.25E-06
rs2062882	8	91839576	G	A	0.587	–0.012	0.002	1.00E-08	0.0083	0.0420	4.21E-01
rs2080870	5	60388313	A	T	0.258	0.012	0.002	4.50E-08	–0.0134	0.0199	2.50E-01
rs215600	7	32333642	G	A	0.358	0.017	0.002	2.00E-15	–0.0015	0.0012	8.87E-01
rs2160896	7	132698885	C	T	0.317	–0.012	0.002	2.20E-08	–0.0038	0.0062	7.32E-01
rs2254710	6	37477000	C	A	0.236	0.013	0.002	3.00E-08	–0.0166	0.0173	1.68E-01
rs2401924	7	115057862	G	C	0.502	0.015	0.002	3.50E-14	–0.0159	0.0141	1.29E-01
rs245774	5	170530930	A	G	0.272	–0.013	0.002	9.00E-09	0.0010	0.0007	9.14E-01
rs2675638	10	63576286	G	A	0.581	0.012	0.002	1.20E-09	–0.0169	0.0134	1.04E-01
rs2678670	2	104469564	A	T	0.485	0.013	0.002	2.80E-10	–0.0016	0.0014	8.73E-01
rs2838834	21	46665208	C	T	0.699	–0.013	0.002	9.20E-10	–0.0159	0.0152	1.48E-01
rs28485305	15	74044197	C	T	0.631	0.011	0.002	2.80E-08	–0.0050	0.0138	6.41E-01
rs2890772	2	146175106	G	T	0.413	–0.02	0.002	3.60E-22	–0.0020	0.0020	8.46E-01
rs2894808	6	52861990	T	A	0.922	–0.022	0.004	4.50E-09	–0.0129	3.0311	5.02E-01
rs317021	4	35418368	T	A	0.814	–0.017	0.003	6.80E-11	0.0059	0.0146	6.58E-01
rs326341	3	107811142	G	A	0.525	0.014	0.002	8.70E-12	–0.0208	0.0124	4.64E-02
rs329120	5	133861756	C	T	0.581	0.014	0.002	5.80E-12	–0.0020	0.0022	8.17E-01
rs348809	20	59032097	A	G	0.348	–0.012	0.002	1.90E-08	–0.0146	0.0155	1.73E-01
rs35120974	5	50977800	A	G	0.583	–0.012	0.002	2.10E-09	0.0034	0.0051	7.47E-01
rs35175834	15	47680815	G	A	0.788	–0.024	0.002	4.80E-22	–0.0080	0.0840	5.38E-01
rs359243	2	60475509	T	C	0.393	–0.013	0.002	8.30E-10	–0.0081	0.0563	4.43E-01
rs369230	16	89645437	G	T	0.308	–0.013	0.002	2.00E-09	–0.0079	0.2041	4.85E-01
rs3742365	14	104198251	T	C	0.595	–0.016	0.002	2.30E-14	0.0126	0.0169	2.29E-01
rs3769949	2	166199284	T	A	0.528	–0.012	0.002	2.90E-09	–0.0040	0.0069	7.19E-01
rs3811038	2	113240183	T	C	0.724	–0.014	0.002	9.20E-10	0.0105	0.0294	3.61E-01
rs421983	3	84892866	T	C	0.519	0.013	0.002	3.10E-10	–0.0227	0.0116	2.50E-02
rs4391802	11	28674592	A	G	0.707	0.015	0.002	1.30E-11	–0.0119	0.0203	2.78E-01
rs4473348	2	182073742	A	T	0.25	–0.015	0.002	9.00E-11	–0.0259	0.0136	2.81E-02
rs4543592	9	3014254	T	C	0.52	–0.012	0.002	4.90E-10	–0.0257	0.0113	1.17E-02
rs4571506	5	87756918	C	T	0.54	0.011	0.002	1.60E-08	–0.0276	0.0113	7.23E-03
rs4671357	2	60136176	T	C	0.519	–0.014	0.002	1.10E-11	–0.0080	0.0536	4.41E-01
rs4814873	20	19616429	C	T	0.767	0.014	0.002	2.70E-09	0.0058	0.0175	6.30E-01
rs4949465	1	32178489	T	C	0.87	–0.017	0.003	1.50E-08	0.0033	0.0035	8.30E-01
rs4957528	5	106420589	A	C	0.208	–0.015	0.002	4.50E-09	0.0070	0.0332	5.83E-01
rs549845	1	44076469	G	A	0.301	0.016	0.002	9.90E-14	–0.0107	0.0260	3.41E-01
rs6011779	20	61984317	C	T	0.191	0.028	0.003	1.60E-27	0.0139	0.0264	2.99E-01
rs60952428	16	75640521	T	C	0.909	0.019	0.003	3.40E-08	–0.0070	0.0117	7.24E-01
rs6119897	20	31145415	G	A	0.762	–0.018	0.002	3.10E-15	0.0104	0.0362	3.88E-01
rs62135536	2	44326028	C	T	0.968	0.035	0.006	6.80E-10	0.0366	0.0448	2.07E-01
rs62155874	2	105973094	A	G	0.873	–0.024	0.003	4.00E-16	0.0014	0.0010	9.29E-01
rs62175972	2	161362830	T	C	0.966	0.031	0.006	1.70E-08	–0.0070	0.0076	8.22E-01
rs624833	4	2881256	T	G	0.695	0.013	0.002	9.20E-10	0.0060	0.0268	5.89E-01
rs6598539	15	99204483	T	C	0.489	–0.012	0.002	3.70E-09	–0.0045	0.0108	6.62E-01
rs6741228	2	22548774	T	C	0.433	0.011	0.002	1.70E-08	–0.0011	0.0008	9.13E-01
rs67596067	17	50333733	G	A	0.649	–0.013	0.002	1.10E-09	–0.0149	0.0148	1.58E-01
rs6778080	3	49317338	T	C	0.267	0.016	0.002	2.10E-12	0.0090	0.0399	4.11E-01
rs6779302	3	16859710	G	T	0.633	–0.013	0.002	1.40E-09	0.0104	0.0235	3.30E-01
rs6935954	6	26255451	A	G	0.421	0.014	0.002	9.60E-12	–0.0284	0.0113	5.99E-03
rs6957896	7	132309592	C	T	0.503	–0.011	0.002	4.60E-08	0.0127	0.0160	2.14E-01
rs6962772	7	99081730	A	G	0.846	0.016	0.003	8.60E-09	–0.0188	0.0213	1.88E-01
rs7039819	9	82430418	G	A	0.427	0.013	0.002	5.20E-10	–0.0061	0.0440	5.55E-01
rs7077678	10	104438565	C	T	0.623	0.012	0.002	2.40E-09	–0.0363	0.0114	7.02E-04
rs71367545	18	77576337	G	A	0.791	–0.015	0.002	1.70E-09	0.0136	0.0245	2.90E-01
rs7144406	14	98659847	A	G	0.791	–0.013	0.002	4.20E-08	0.0220	0.0159	8.25E-02
rs7155595	14	77502546	A	C	0.674	–0.013	0.002	3.40E-09	0.0124	0.0190	2.57E-01
rs72678864	4	112422145	G	A	0.829	0.018	0.003	1.70E-11	–0.0159	0.0218	2.34E-01
rs7297175	12	56473808	T	C	0.431	–0.012	0.002	1.10E-08	0.0639	0.0105	6.52E-10
rs732083	17	37834367	G	A	0.333	0.012	0.002	1.40E-08	0.0198	0.0135	7.09E-02
rs73220544	3	131074511	A	C	0.842	–0.016	0.003	1.50E-08	–0.0109	0.1011	4.57E-01
rs7333559	13	100546450	G	A	0.212	0.015	0.002	5.30E-10	–0.0162	0.0199	2.07E-01
rs74086911	12	50015942	G	A	0.925	0.021	0.004	2.00E-08	–0.0266	0.0274	1.65E-01
rs7528604	1	66407352	G	A	0.566	0.014	0.002	6.60E-12	–0.0100	0.0248	3.44E-01
rs7553348	1	75005067	G	A	0.438	0.014	0.002	5.90E-12	0.0030	0.0035	8.06E-01
rs7569203	2	45154418	A	C	0.689	–0.016	0.002	5.50E-13	0.0082	0.0877	4.63E-01
rs75742406	11	17070365	G	A	0.739	0.014	0.002	1.20E-09	–0.0060	0.0288	5.82E-01
rs76608582	19	4474725	C	A	0.953	0.031	0.005	3.40E-10	–0.0334	0.0330	1.56E-01
rs775758	3	77582005	A	T	0.433	0.011	0.002	1.20E-08	–0.0015	0.0012	8.87E-01
rs7766610	6	111707821	C	A	0.183	0.018	0.003	2.40E-12	0.0090	0.1514	4.76E-01
rs7775552	6	67475569	A	G	0.513	–0.012	0.002	2.50E-09	–0.0030	0.0035	8.03E-01
rs7807019	7	117543063	A	G	0.54	–0.015	0.002	7.50E-14	0.0093	0.0262	3.61E-01
rs7897434	10	74733767	G	C	0.633	0.012	0.002	2.40E-08	–0.0100	0.0256	3.49E-01
rs8042134	15	97514404	T	G	0.541	–0.014	0.002	2.20E-12	–0.0109	0.0206	2.97E-01
rs8042849	15	78817929	C	T	0.342	0.028	0.002	2.40E-39	–0.0202	0.0131	6.17E-02
rs860326	14	57342912	C	T	0.428	0.012	0.002	3.20E-09	0.0129	0.0152	1.98E-01
rs8614	17	27588806	C	A	0.818	–0.017	0.003	1.50E-10	–0.0227	0.0161	7.92E-02
rs889398	16	69556715	C	T	0.588	0.013	0.002	3.90E-11	–0.0129	0.0162	2.13E-01
rs912780	13	67336339	T	G	0.65	0.012	0.002	8.50E-09	–0.0178	0.0143	1.06E-01
rs9435340	1	107593201	T	A	0.344	0.012	0.002	1.00E-08	–0.0145	0.0160	1.83E-01
rs9842947	3	157412246	C	T	0.326	–0.012	0.002	4.40E-09	–0.0072	0.2826	5.10E-01
rs986391	5	166993972	G	A	0.367	0.016	0.002	1.30E-14	–0.0070	0.2547	5.11E-01
rs9904288	17	47031973	T	C	0.708	0.012	0.002	2.40E-08	–0.0020	0.0020	8.37E-01
rs9919670	11	112877304	G	A	0.612	–0.022	0.002	2.10E-26	0.0072	0.4060	4.93E-01

*Beta is the regression coefficients based on the effect allele. EA, effect allele; NEA, non-effect allele; EAF, effect allele frequency; GWAS, genome-wide association studies; and SE, standard error.*

### Asthma Genome-Wide Association Study Dataset

The asthma GWAS dataset from the UK Biobank included 487,409 participants. Before the GWAS analysis, the data were filtered by excluding non-white individuals, excluding individuals with missing genotype information, limiting to doctor-diagnosed asthma cases, and excluding individuals without complete phenotype or covariate information (Zhu et al., 2018). Finally, a total of 14,085 asthma participants and 76,768 controls were selected for the GWAS analysis (Zhu et al., 2018). The detailed information about the age and sex characteristics is provided in Table 2, as described in the original study (Zhu et al., 2018).

**TABLE 2:** Age and sex characteristics of the UK Biobank asthma and control subjects.

Phenotypes	*n*	Male (*n*, %)	Age, years (mean, SD)
All asthma	14,085	6,191 (44.0%)	56.27 (8.10)
Non-allergic asthma	7,908	3,697 (46.8%)	57.20 (7.98)
Allergic asthma	6,177	6,175 (40.4%)	55.07 (8.09)
Controls	76,768	37,400 (48.7%)	57.23 (7.86)

### Pleiotropy Analysis

We conducted a pleiotropy analysis to assure that all the selected 124 genetic variants associated with lifetime smoking met the MR assumptions, as has been done in previous studies (Cheng et al., 2018, 2019; Liu et al., 2018; Luo et al., 2019; Zhao and Schooling, 2019; Zhao et al., 2019). Here, we selected two different methods. First, we used the MR–Egger intercept test to detect the presence of potential pleiotropy, and more detailed information about MR–Egger intercept test has been provided in previous studies (Cheng et al., 2018, 2019; Liu et al., 2018). Meanwhile, we selected MR pleiotropy residual sum and outlier (MR-PRESSO) test to perform the pleiotropy analysis (Verbanck et al., 2018). There are three different tests in MR-PRESSO. MR-PRESSO global test could detect horizontal pleiotropy (Verbanck et al., 2018). The significance level for the pleiotropy analysis was set as *P* < 0.05.

### Mendelian Randomization Analysis

For the MR analysis, we used the inverse variance weighted (IVW) meta-analysis; and for the sensitivity analysis, we used both the weighted median regression method and MR–Egger method, as has been done in previous studies (Liu et al., 2018; Luo et al., 2019; Zhao and Schooling, 2019; Zhao et al., 2019). Even if half of the 124 genetic variants did not meet the MR assumptions, the weighted median regression method can still produce a consistent estimate of the causal association (Emdin et al., 2017). If there is significant pleiotropy, the MR–Egger method can adjust the pleiotropy and produce a corrected estimate of the causal association (Bowden et al., 2015, 2016; Dale et al., 2017; Tillmann et al., 2017; Yavorska and Burgess, 2017). Meanwhile, MR-PRESSO method was also selected as the sensitivity analysis. MR-PRESSO outlier test could correct for horizontal pleiotropy via outlier removal. MR-PRESSO distortion test could evaluate the differences in the causal estimates before and after correction for outliers (Verbanck et al., 2018). All the statistical analyses were conducted using R (v3.2.4) and R package “MendelianRandomization” and MR-PRESSO (Cheng et al., 2018, 2019; Liu et al., 2018). The significance threshold for the MR analysis was set as *P* < 0.05. Finally, a leave-one-out permutation analysis was used to evaluate the influence of each instrumental variable on the MR estimate (Liu et al., 2018).

## Results

### Genome-Wide Association Study Summary Statistics

Of the 124 genetic variants from the lifetime smoking GWAS dataset, only 117 were included in the asthma GWAS dataset. The missing variants were rs2867112, rs3896224, rs11768481, rs35169606, rs13016665, rs62098013, and rs35343344. Summary statistics of the 117 genetic variants in the lifetime smoking and asthma GWAS datasets are provided in Table 1. These 117 genetic variants were used in the subsequent analysis.

### Pleiotropy Analysis

The pleiotropy analysis using the MR–Egger intercept test showed that all 117 genetic variants had no significant pleiotropy (intercept = 0.001, *P* = 0.187). Hence, evidence from MR–Egger intercept test supported that all 117 genetic variants could be taken as the potential instrumental variables. However, MR-PRESSO global test indicated significant horizontal pleiotropy *P* = 0.003. Hence, we further correct for the horizontal pleiotropy via outlier removal using MR-PRESSO outlier test.

### Mendelian Randomization Analysis

**FIGURE 1:**
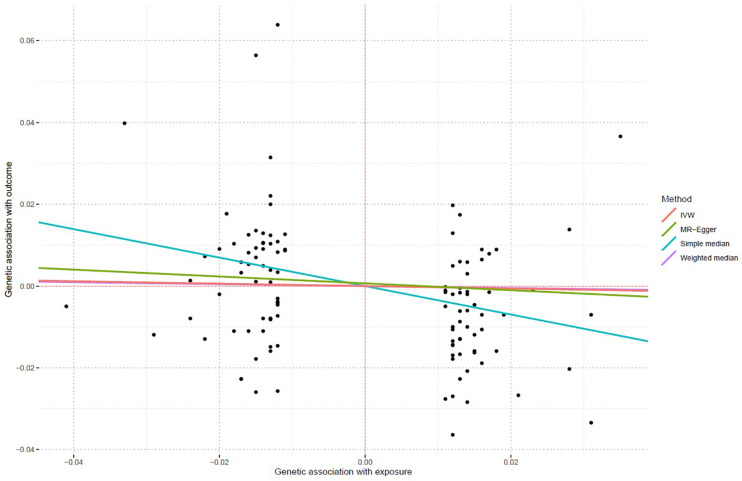
Individual causal estimates from each of 117 genetic variants using different methods. This scatter plot shows individual causal estimates from each of 117 genetic variants associated with lifetime smoking on the *x*-axis and asthma risk on the *y*-axis. The continuous line represents the causal estimate of lifetime smoking on asthma risk.

Using all 117 genetic variants, we found that increased lifetime smoking was significantly associated with decreased asthma risk using both the IVW method (OR = 0.971, 95% CI 0.956–0.986, and *P* = 1.77E-04) and the weighted median regression method (OR = 0.976, 95% CI 0.96–0.994, and *P* = 8.00E-03). The MR–Egger method also showed that lifetime smoking reduced the risk of asthma (OR = 0.919, 95% CI 0.847–0.998, *P* = 4.5E-02). Importantly, all three methods produced consistent results. Figure 1 shows the individual causal estimates from each of the 117 genetic variants using the different methods.

With the use of all 117 genetic variants, MR-PRESSO MR analysis also indicated a significant association between increased lifetime smoking and decreased asthma risk with OR = 0.971, 95% CI 0.956–0.986, and *P* = 2.69E-04. After the outlier was removed, MR-PRESSO outlier test further supported the significant association with OR = 0.971, 95% CI 0.959–0.984, and *P* = 1.57E-05. Importantly, there is no significant difference in the causal estimates before and after correction for outliers (*P* = 0.925).

**FIGURE 2:**
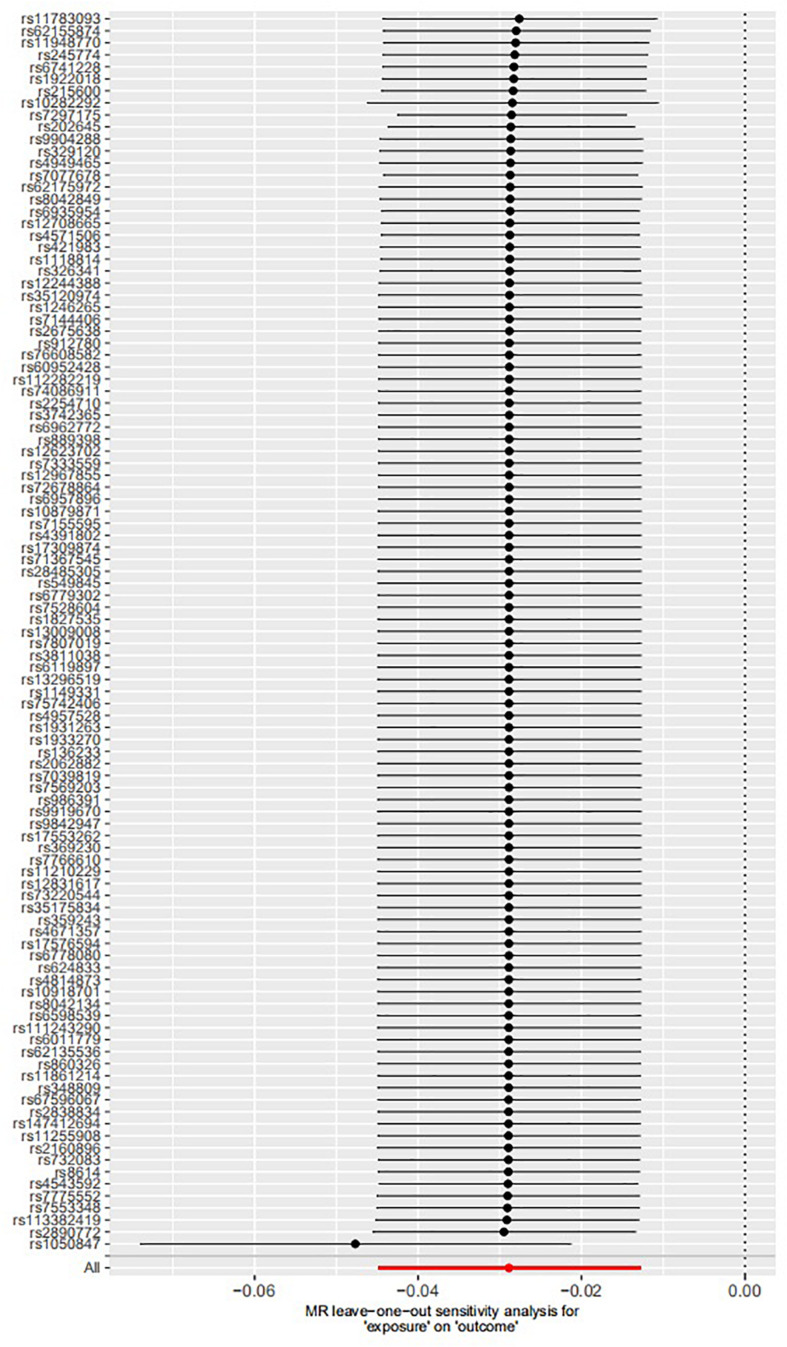
Influence of each instrumental variable on the Mendelian randomization (MR) estimate using inverse variance weighted (IVW) method. Only one single genetic variant rs1050847 could significantly affect the estimates between increased lifetime smoking and decreased asthma risk using IVW method.

**FIGURE 3:**
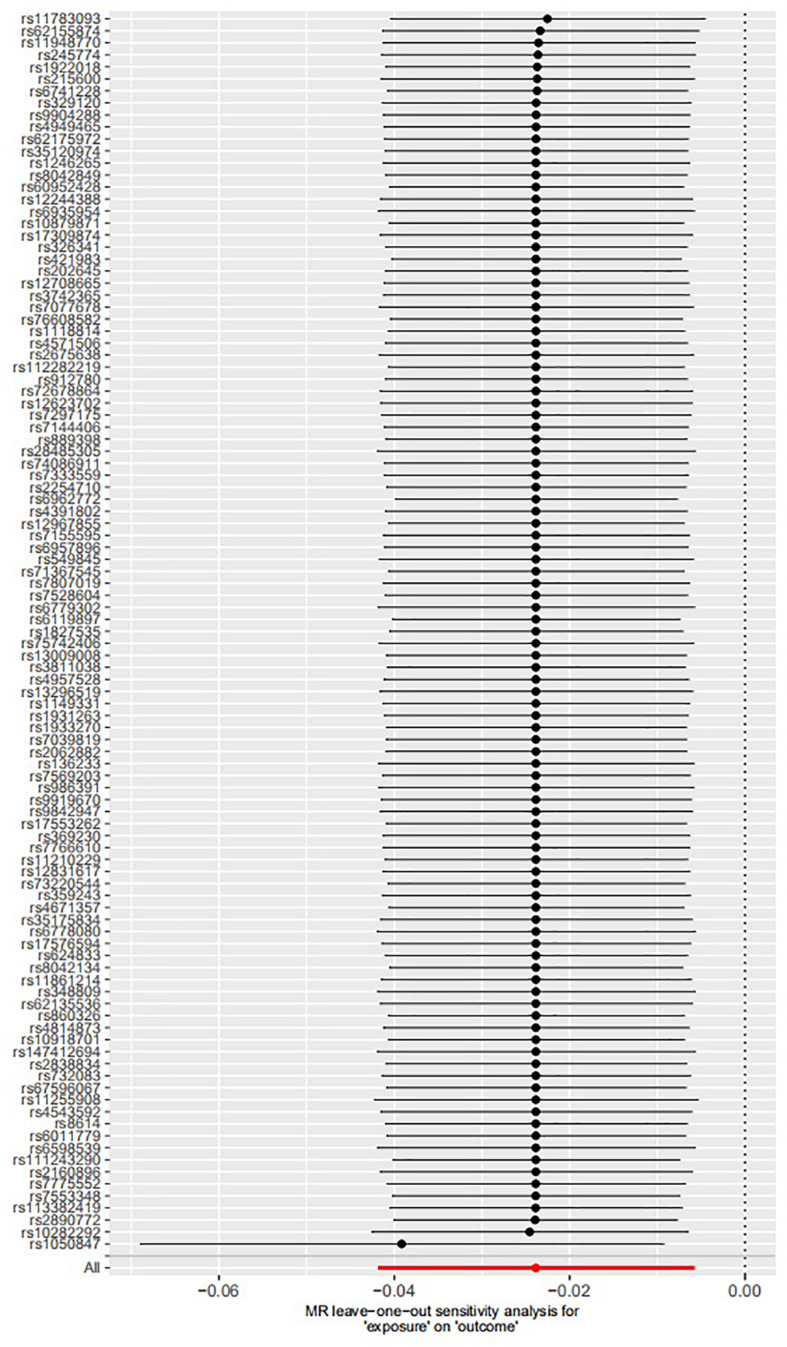
Influence of each instrumental variable on the Mendelian randomization (MR) estimate using weighted median regression method. Only one single genetic variant rs1050847 could significantly affect the estimates between increased lifetime smoking and decreased asthma risk using weighted median regression method.

**FIGURE 4:**
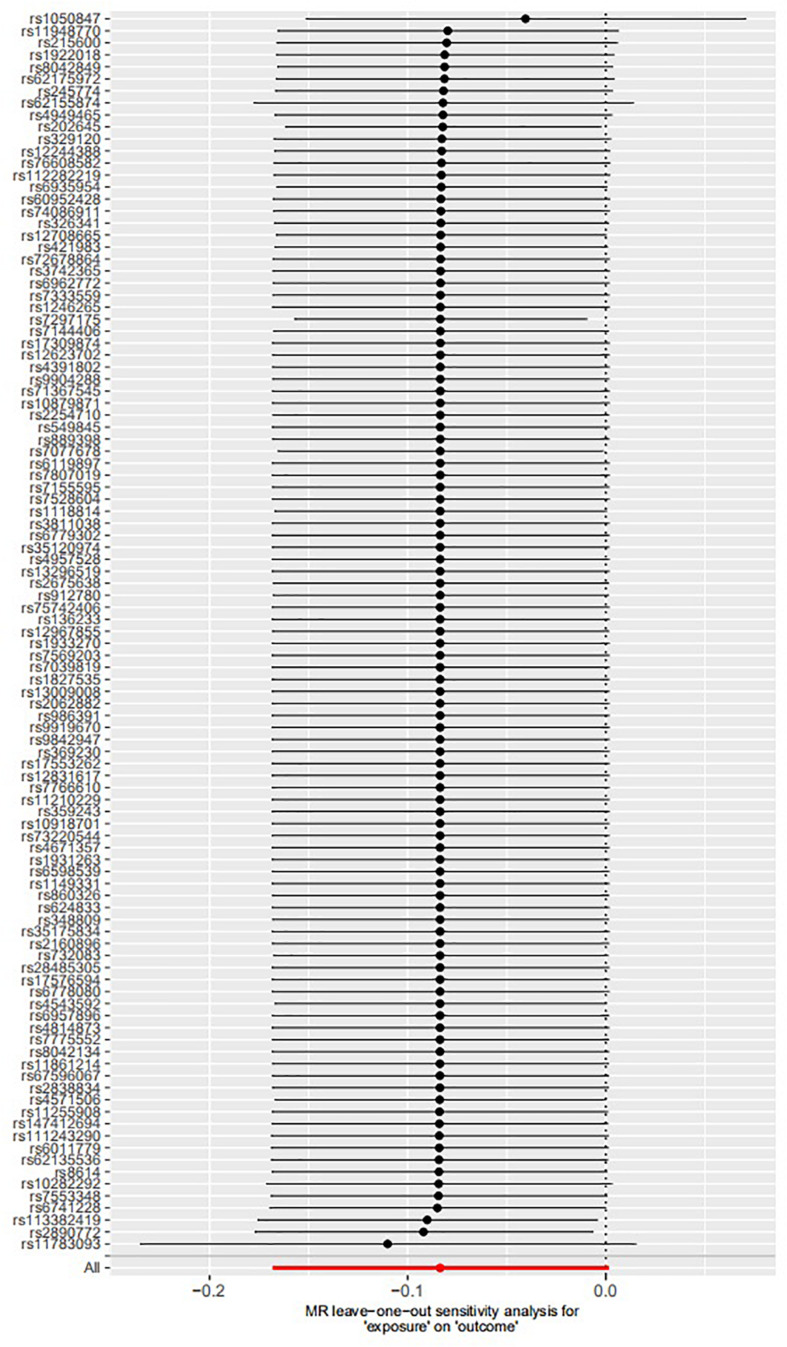
Influence of each instrumental variable on the Mendelian randomization (MR) estimate using MR–Egger method. Only one single genetic variant rs1050847 could significantly affect the estimates between increased lifetime smoking and decreased asthma risk using MR–Egger method.

The leave-one-out permutation showed that only one single genetic variant rs1050847 could significantly affect the estimates between increased lifetime smoking and decreased asthma risk. Figures 2–4 show the influence of each instrumental variable on the MR estimate using IVW method, weighted median regression method, and MR–Egger method, respectively.

## Discussion

Epidemiological studies indicate that asthma and allergic disease co-occur and share a similar and very close course especially in the children (Ober and Yao, 2011; Guibas et al., 2017; Schoettler et al., 2019). It is reported that allergic asthma is most common in kinds of diseases in childhood (Cookson and Moffatt, 2000). It is known that both asthma and allergic disease are complex human diseases, which are caused by both genetic and environmental contributions (Ober and Yao, 2011; Guibas et al., 2017).

Until recently, large-scale GWAS and meta-analyses of GWAS have been conducted and identified common genetic variant, risk genes, or pathways that contribute to both diseases (Ferreira et al., 2017; Zhu et al., 2018). In a recent GWAS, Ferreira et al. analyzed 360,838 samples and demonstrated common genetic variants to be shared by asthma, hay fever, and eczema (Ferreira et al., 2017). Importantly, these risk variants could regulate and dysregulate the expression of immune-related genes (Ferreira et al., 2017). In 2018, Zhu et al. (2018) analyzed 33,593 cases and 76,768 controls and identified a positive genetic correlation between asthma and allergic diseases.

However, Skaaby et al. (2017) found that smoking was significantly associated with increased risk of asthma, reduced hay fever, and reduced allergic sensitization risk by an MR analysis. These findings are inconsistent, and currently, no consensus exists. In their MR analysis, Skaaby et al. (2017) selected only one single genetic variant rs16969968 as the instrumental variable. It is reported that MR studies that use a single genetic variant as the instrumental variable have some limitations (Relton and Davey Smith, 2015; Davies et al., 2018; Rosa et al., 2019). Hence, an MR analysis using multiple significant and independent genetic variants as potential instrumental variables may increase the statistical power and precision (Relton and Davey Smith, 2015; Davies et al., 2018; Rosa et al., 2019).

Here, we conducted an updated MR analysis to investigate the causal association between lifetime smoking and asthma risk using the 124 genetic variants from the lifetime smoking GWAS dataset as the instrumental variables (Wootton et al., 2019). We first performed a pleiotropy analysis using the MR–Egger intercept test, and we found no significant pleiotropy. We further conducted a pleiotropy analysis using MR-PRESSO global test, and we found significant pleiotropy (Verbanck et al., 2018). In the MR analysis, we first selected three methods including IVW, weighted median regression, and MR–Egger methods, and we clearly demonstrated that increased lifetime smoking was significantly associated with decreased asthma risk. We further conducted an MR analysis using MR-PRESSO method and verified the significant association between increased lifetime smoking and decreased asthma risk. MR-PRESSO outlier test further supported the significant association after removing the outlier. Importantly, no significant difference was observed in the causal estimates before and after correction for outliers. Finally, the leave-one-out permutation showed that only one single genetic variant rs1050847 could significantly affect the estimates between increased lifetime smoking and decreased asthma risk.

Our results are consistent with the results from a large-scale population-based international cohort study by Cerveri et al. (2012) who analyzed 9,092 non-asthma and 1,045 asthma samples. They found that the proportion (26%) of smokers in the asthma group was significantly less than the proportion (31%) of smokers in the non-asthma group (*P* < 0.001; Cerveri et al., 2012). In the asthma group, smoking increased the mean asthma score (Cerveri et al., 2012). A study using mouse mast cells showed that cigarette smoke prevented allergies by decreasing the reaction of immune cells to allergens (Mortaz et al., 2009).

## Data Availability Statement

All datasets presented in this study are included in the article/supplementary material.

## Author Contributions

HZ designed the project. MS analyzed the data. All authors contributed to the first draft of the manuscript and read and approved the final manuscript.

## Conflict of Interest

The authors declare that the research was conducted in the absence of any commercial or financial relationships that could be construed as a potential conflict of interest.
